# The female reproductive tract-gut axis in gastrointestinal parasitic infection

**DOI:** 10.1371/journal.ppat.1013711

**Published:** 2025-12-03

**Authors:** Olivia Shorthouse, Alice H. Costain, Andrew S. MacDonald, Juan F. Quintana

**Affiliations:** 1 Lydia Becker Institute of Immunology and Inflammation, University of Manchester, Manchester, United Kingdom; 2 Division of Immunology, Immunity to Infection and Respiratory Medicine, University of Manchester, Manchester, United Kingdom; 3 Institute of Immunology and Infection Research, University of Edinburgh, Edinburgh, United Kingdom; 4 Geoffrey Jefferson Brain Research Centre, University of Manchester, Manchester, United Kingdom; University of Wisconsin-Madison, UNITED STATES OF AMERICA

## Abstract

Mucosal barrier sites are specialised interfaces that constantly defend against challenges, from tissue damage to infections. When considering these sites, we typically think of the lungs, gut, mouth or skin. However, a site often overlooked is the female reproductive tract (FRT), an equally dynamic mucosal site uniquely tasked with both immune defence and sustaining new life. The FRT undergoes dramatic tissue remodelling and can return to a homeostatic state without scarring – an ability shared only with the oral mucosa and foetal skin. Given the interconnected nature of mucosal systems, disruption of intestinal homeostasis by enteric or systemic infection has broad impacts on systemic immunity, prompting us to consider how such disturbances could impact the FRT.

## Homeostatic FRT immune microenvironment

**Fig 1:**
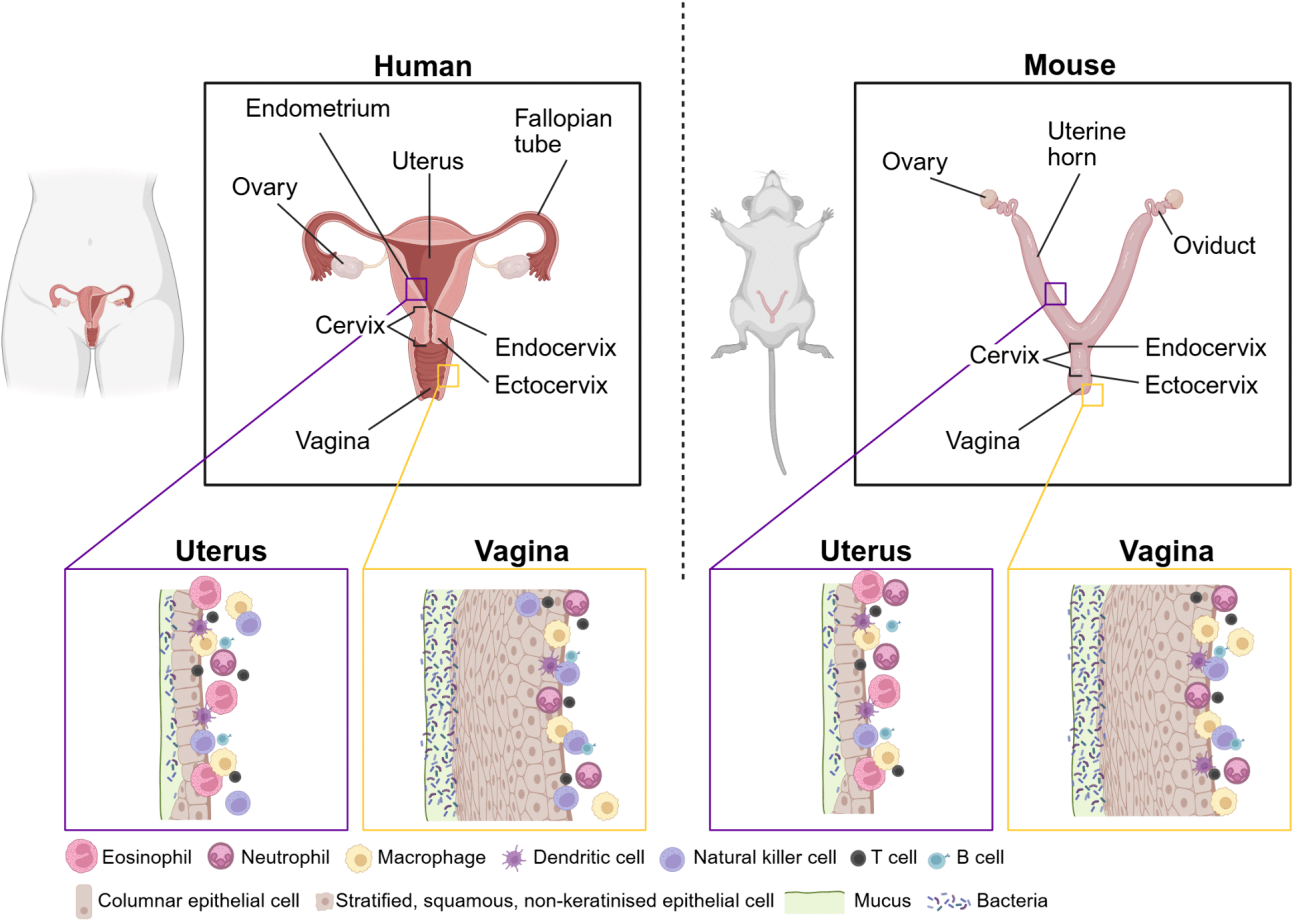
Anatomy and immune landscape of the female reproductive tract (FRT) in humans and mice. The FRT in both species includes the ovaries, oviducts/fallopian tubes, uterus, cervix (endocervix and ectocervix), and vagina. Notable anatomical differences exist where humans have a single, pyriform uterus connected to the ovaries by long fallopian tubes, whereas mice possess a bicornuate uterus (two uterine horns) with short oviducts. In both species, the upper FRT is lined by a monolayer of columnar epithelial cells, which transitions at the cervical junction to stratified, squamous, non-keratinised epithelium in the lower FRT. Across both regions, a variety of innate and adaptive immune cells contribute to immune surveillance while preserving tissue homeostasis [7,40,41]. Additional defence mechanisms, including mucus, antimicrobial peptides (AMPs), and resident microbiota, support this immune balance. Created in BioRender. Shorthouse, O. (2025) https://BioRender.com/1xl8eue.

In both humans and mice, the FRT can be divided into upper and lower regions, which encounter different challenges and so have different cellular and structural compositions [1]. The lower FRT (vagina and ectocervix) is lined by a protective stratified, squamous, non-keratinised epithelium, which contains mucin-secreting cells. Similar to other mucosal barrier sites, this mucus plays a role in pathogenic defence, but is also important in reproductive success [2,3]. In contrast, the upper FRT (ovaries, fallopian tubes, uterus and endocervix) is covered by a monolayer of columnar epithelial cells containing both ciliated and mucin-secretory cells, resembling the structure of the intestinal epithelium [4–6] (Fig 1).

While the general FRT features are conserved across species, the regulation of menstrual/oestrus cycles, litter size, uterine and placental structure can differ between mammalian species (e.g., humans have a single, pear-shaped uterus, whereas mice possess a bicornuate uterus to accommodate large litters) [7] (Fig 1). FRT immune defence relies on tight coordination between mechanical barriers, chemical secretions and dynamic immune cell populations [1,4]. In the lower FRT, a controlled immune response is critical for pathogen defence and prevention of infections moving upwards to the uterus, particularly against sexually transmitted infections (STIs) like *Chlamydia trachomatis* and herpes simplex virus (HSV)-2 [8]. In contrast, a more tolerogenic upper FRT environment is important to accommodate reproductive functions, particularly during stages of repair as part of the menstrual/oestrous cycles and for successful pregnancy maintenance [9].

Above all and across species, the FRT immune landscape is tightly regulated by hormonal fluctuations across menstrual/oestrous cycles [3]. Circulating sex steroid hormones, particularly oestrogen and progesterone, influence structural and immune cells, shaping infection susceptibility based on menstrual cycle stage, pregnancy status or hormonal contraceptive use [10–12]. For example, progesterone can thin the vaginal epithelium, weakening its barrier function and increasing vulnerability to pathogens [10]. Sex hormones also regulate the composition and viscosity of cervical mucus, which plays an important role in resistance to STIs [10]. These hormonal shifts dynamically reshape local immune environments by modulating abundance and function of specific immune cell subsets, which can either promote protective responses or increase local infection susceptibility. Dysregulation of the FRT immune landscape has been implicated in reproductive health disorders in women and inadequate protection against pathogens [13–15]. Nevertheless, the specific immunological mechanisms underlying this dysregulation, and the extent to which systemic immune changes contribute to local FRT responses, remain unclear.

**Fig 2:**
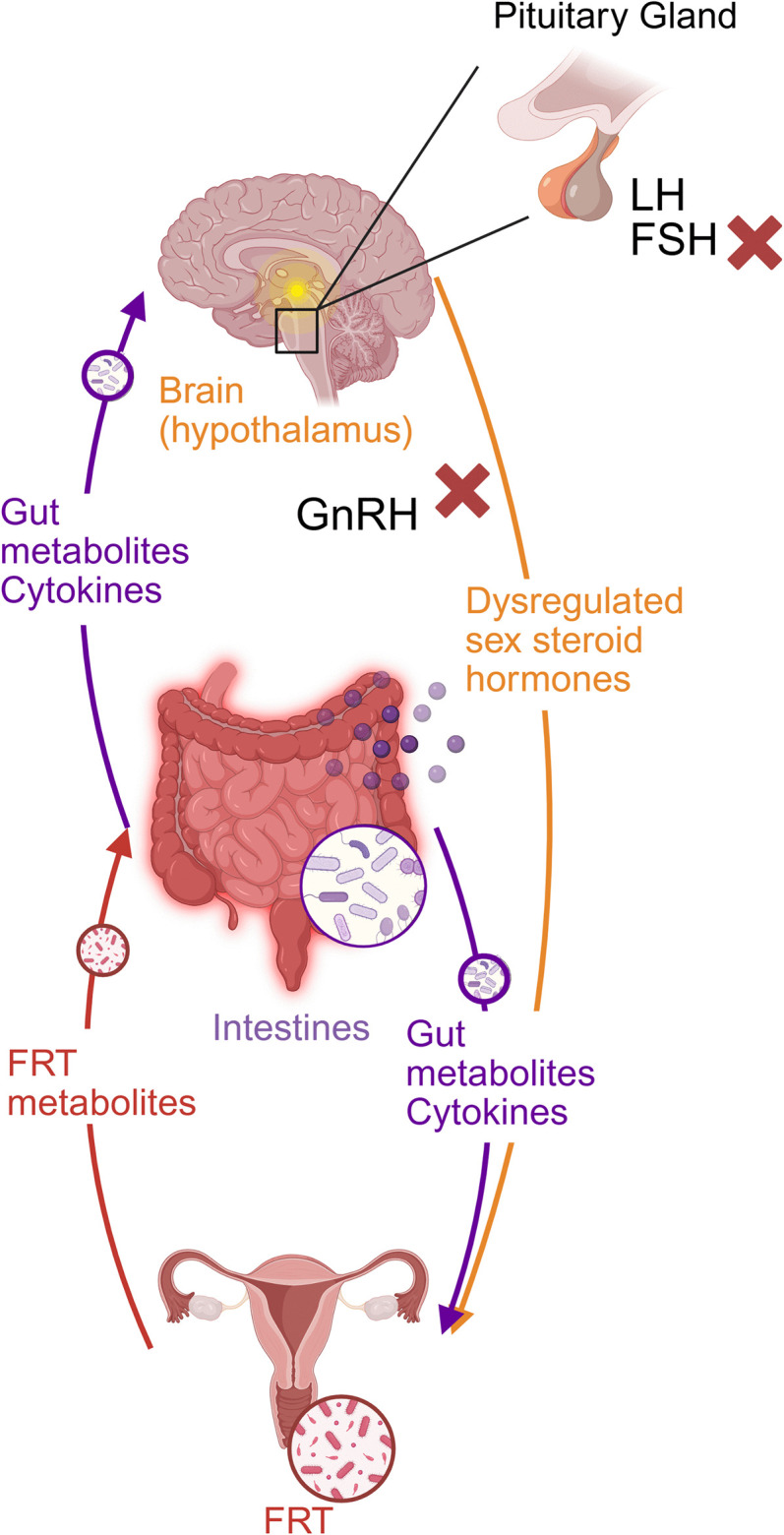
Disruption of gut-brain FRT crosstalk during inflammation or infection. Diagram illustrating potential routes of communication between the gut, brain and FRT that may influence the FRT immune landscape. Cytokines and gut-derived metabolites can signal to the brain, potentially suppressing hypothalamic gonadotropin-releasing hormone (GnRH) production and downstream luteinising hormone (LH) and follicle-stimulating hormone (FSH) release from the pituitary gland [34]. This suppression leads to dysregulated sex steroid hormone levels, which will impact the FRT immune landscape and reproductive function [36]. In parallel, gut-derived metabolites and cytokines may directly act on the FRT to alter its function [30]. Conversely, FRT-derived metabolites may feed back to modulate gut homeostasis [32]. Created in BioRender. Shorthouse, O. (2025) https://BioRender.com/8gd9qy3.

While the FRT immune landscape is shaped by intrinsic factors (including hormonal fluctuations), systemic factors, including those originating from the gut, play a significant role in modulating the FRT immune microenvironment [3,16]. The intestines are increasingly regarded as a central immunological organ, capable of bidirectional communication across a range of other mucosal barrier sites, including the FRT [16,17]. This communication can involve microbial metabolites, cytokines, and hormonal modulation (Fig 2). As such, the gut-FRT axis emerges as a critical yet understudied contributor to female reproductive health and FRT homeostasis [18]. Here, we highlight evidence linking gut homeostasis to FRT health and present potential hypotheses to inform future research.

## GI parasitic infections and the FRT

Epidemiological and clinical studies suggest links between helminth exposure and fecundity changes in infected women [19]. Indeed, almost a decade of data from Amazonian women associated hookworm helminth infections with alterations in female fecundity, including delayed first pregnancy and reduced likelihood of successive pregnancy [19]. Other reports indicate that *Heligomosomoides bakeri* and *Ancylostoma duodenale* intestinal infections negatively affect pregnancy outcomes in women [20,21]. Although helminth-induced anaemia may contribute to these effects, these data led the World Health Organisation to recommend anthelmintic treatment for women of childbearing age in endemic regions [22]. Similarly, in rodent studies, maternal helminth infections are associated with reduced foetal weight [23], reduced reproductive efficiency [24], and fewer surviving pups [25].

Helminth-induced immune alterations are further implicated in susceptibility to STIs, impacting current public health efforts to control and eradicate STIs [26]. Soil-transmitted helminth infections localised away from the FRT in the GI tract are associated with increased human papilloma virus prevalence in older women, with cytokine analysis of vaginal lavages suggesting a role for elevated IL-4 [27]. A recent study in mice exploring potential immunological bases for geographical overlap between helminth infection and STIs showed that prior helminth infection worsened HSV-2 pathology [28]. Despite these observations, further insight into the pathways linking distal helminth infections to FRT immunological changes is needed. This relationship could be mediated by the microbiota, parasite manipulation of the immune system and neuroimmune interactions.

## Potential drivers of FRT-Gut crosstalk during GI infections

### The microbiome

Helminth infections are known to reshape the gut microbiome resulting in dysbiosis [29]. Given that the gut microbiome is increasingly recognised as a key influencer of FRT immunology [16,18], helminth-induced dysbiosis may have far-reaching effects on the FRT homeostasis. Gut dysbiosis can disrupt oestrogen homeostasis by deconjugating oestrogen metabolites and facilitating reabsorption into circulation [30]. These hormonal imbalances dysregulate the FRT immune landscape, which is highly responsive to hormonal fluctuations [31]. Importantly, this is not limited to the infection context, as increased levels of *Gardnerella vaginalis* (associated with bacterial vaginosis) are detected in patients with inflammatory bowel disease [32], further demonstrating a potential link between intestinal health and FRT homeostasis. Gut microbiome perturbations have also been implicated in pregnancy-related reproductive disorders, including intrauterine growth restriction and preterm birth [33]. Another route of FRT disruption is through gut microbiome-derived metabolites, particularly short-chain fatty acids, which can modulate the host’s neuronal network and the hypothalamus-pituitary gland-gonadal (HPG) axis [34]. Given that intestinal helminth infections can alter both microbial composition and metabolite production [35], it is likely that helminth-driven immune responses and hormonal alterations could contribute to FRT dysfunction.

## Parasite- and immune-derived mediators

Helminths are well known for their ability to modulate systemic immunity [17]. While some of these modulations confer host benefits, including dampening excessive inflammation, they can also be detrimental. Balanced coordination of type 1, type 2, and type 17 immunity is paramount to support FRT functions such as menstruation, implantation and pregnancy while still maintaining effective pathogen defences [4]. However, helminth ability to skew responses towards type 2 immunity (primarily via cytokines such as IL-4, IL-5, and IL-13) potentially compromises the FRT immune microenvironment. Dysregulation of this balance may weaken mucosal defence, increasing susceptibility against potentially harmful pathogens. Indeed, *Nippostrongylus brasiliensis* intestinal infection in mice expanded eosinophils and group 2 innate lymphoid cells in the lower FRT, generating a type 2-dominated immune landscape that exacerbated HSV-2 pathology in an IL-5-dependent manner [28]. This underscores the need to explore how helminth infections reshape FRT immunity, particularly in the upper reproductive tract, where immune and hormonal disruptions have the potential to compromise barrier integrity with long-term consequences for fertility.

## Neuroimmune interactions

While helminths directly skew systemic immunity, their influence extends beyond immunological factors with systemic responses potentially disrupting reproductive function through neuroimmune pathways. Several shared receptors and mediators exist between cytokine signalling and the HPG axis, the main regulator of vertebrate reproduction [36]. For example, the proinflammatory cytokine TNF-α can inhibit the release of gonadotropin-releasing hormone from the hypothalamus, thereby impairing reproductive function [37], but the impact of inflammation on hormone receptors in other sites (e.g., pituitary gland) remains elusive. Given the magnitude and chronicity of immune modulation during helminth infection, it is plausible that systemic inflammation and/or altered enteric nervous system signalling could interfere with hypothalamic function and downstream pituitary-gonad communication. These biologically plausible interactions could shed mechanistic light on crosstalk between immunocompetence and reproductive capacity. Could the reproductive suppression and increased STI vulnerability observed in helminth-infected women reflect an immune trade-off - one that prioritises systemic immunity at the cost of local FRT integrity?

## Outlook and future directions

Helminth infections exert dramatic immunological effects throughout the body, which can help or hinder the immune response to secondary infections. The same applies to other parasitic diseases that have a direct or indirect effect on the GI tract, including malaria [38], American Trypanosomiasis [39] and potentially Human African Trypanosomiasis. These effects have been extensively explored in the lung, gut, and skin, but there are very few studies assessing this in the FRT. Therefore, understanding how helminths and other parasites alter this unique mucosal barrier site is essential to better understand women’s reproductive health, especially in areas where these infections disproportionately affect women and children.
